# Erratum

**Published:** 1996

We regret that an error occurred in the colorizing of figure 6 in the article “A Primer on Imaging,” by John J. Doria, and figure 1 in the article “Measuring Electrical Activity of the Brain: ERP Mapping in Alcohol Research,” by David B. Chorlian, Bernice Porjesz, and Howard L. Cohen, in volume 19, number 4, 1995, pages 264 and 316, respectively. The corrected figure and the full figure legend appear below.

**Figure:**
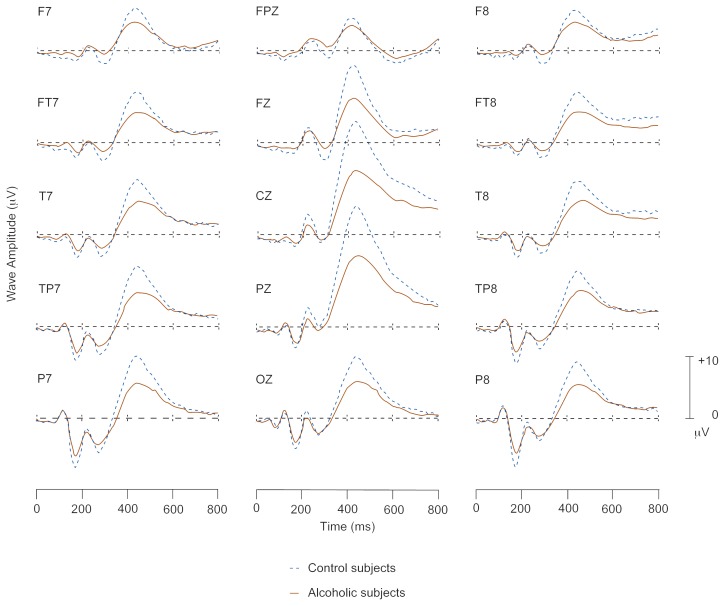
Electroencephalographic (i.e., brain wave) tracings of the waveform P300 obtained from nonalcoholic (i.e., control) and alcoholic subjects in response to a visual stimulus. Each tracing represents a different location on the scalp. The horizontal scales represent time in milliseconds (ms). The vertical scales represent wave amplitude measured in microvolts (μV) of electrical potential. The data are statistically derived from 197 subjects and 61 scalp electrodes.

